# Research on Deployable Wings for MAVs Bioinspired by the Hind Wings of the Beetle *Protaetia brevitarsis*

**DOI:** 10.3390/biomimetics9060313

**Published:** 2024-05-23

**Authors:** Jiyu Sun, Wenzhe Wang, Pengpeng Li, Zhijun Zhang

**Affiliations:** 1 Key Laboratory of Bionic Engineering (Ministry of Education, China), Jilin University, Changchun 130022, China; sjy@jlu.edu.cn (J.S.); Wangwz22@mails.jlu.edu.cn (W.W.); lipengpengiii@163.com (P.L.); 2 Key Laboratory of CNC Equipment Reliability (Ministry of Education), School of Mechanical and Aerospace Engineering, Jilin University, Changchun 130022, China

**Keywords:** beetle, hind wing, bionic deployable wing (BD-W), wind tunnel, micro aerial vehicles (MAVs)

## Abstract

Deployable hind wings of beetles led to a bio-inspired idea to design deployable micro aerial vehicles (MAVs) to meet the requirement of miniaturization. In this paper, a bionic deployable wing (BD-W) model is designed based on the folding mechanism and elliptical wing vein structure of the *Protaetia brevitarsis* hindwing, and its structural static and aerodynamic characteristics are analyzed by using ANSYS Workbench. Finally, the 3D-printed bionic deployable wing was tested in a wind tunnel and compared with simulation experiments to explore the effects of different incoming velocity, flapping frequency, and angle of attack on its aerodynamic characteristics, which resulted in the optimal combination of the tested parameters, among which, the incoming velocity is 3 m/s, the flapping frequency is 10 Hz, the angle of attack is 15°, and the lift-to-drag ratio of this parameter combination is 4.91. The results provide a theoretical basis and technical reference for the further development of bionic flapping wing for MAV applications.

## 1. Introduction

Wings play a critical role in the flight of insects. When beetles (a collective term for Coleoptera insects) are not flying, their hind wings can be folded to reduce their size to hide under the sheath wings to avoid damage [1]. Also, due to their small size and light weight, beetles have become ideal mimics for micro air vehicles (MAVs) [2].

Based on the deployable hind wings of beetles, researchers have developed several folding/expanding mechanisms with high folding rates [3,4,5]. The folding modes of the hind wings of beetles are mainly divided into single-node folding, multi-node folding [6], and sector folding [3]. Different beetles have different folding patterns and folding processes but still follow the multi-node folding method, and their folding behavior is determined by the distribution of wing veins and folding lines [6]. As an increasing number of researchers attach importance to the development of flapping wing MAV and devote themselves to relevant research topics, great progress has been made in the study of bionic deployable wings [7,8]. A low-cost bionic wing fabrication method was proposed by observing the hind wings of A. dichotoma [9]. The fabrication method allows for the free customization of the plane geometry and venation structure of the bionic wing so that its structural parameters can be qualitatively studied. However, the material mechanical properties of the bionic wing produced by this research method are not ideal and need to be further optimized by selecting suitable materials. Truong designed a bionic deployable wing based on a linkage mechanism by taking the hind wing of A. dichotoma as the prototype [10]. The structure of the folding wing is simple and easy to process, but it relies on external forces to achieve transmission and folding, and the wing cannot control and adjust its shape in time during flapping. A bionic flapping wing structure designed by imitating the folding mechanism of C. buqueti hind wings, which transfers the telescopic motion at the wing root to the rotational motion at the hinge, can enable the folding wing to achieve a high folding rate [11], but the folding mechanism is not supported by a support rod, and the wing is easily deformed during high-speed movements. Inspired by the beetle *P. Marginata* [3], Dufour [12] designed a bionic folding wing based on the four-fold knot origami theory established by Hass. The folding scheme verified its feasibility for implementation on MAV through the design and fabrication of origami wings, but the proposed thick origami structure relied on manual assembly and the fabrication process was complicated compared with other origami fabrication techniques, and the process needed to be improved.

When researchers conduct structural static analysis of the foldable hind wings of beetles, their wing veins are usually simplified, and a three-dimensional coupled model of the hind wings is established for finite element simulation mechanical analysis. It was found that the wing veins are the main load-bearing part of the hind wings and play a great role in supporting and relieving stress, while the structure of the wing veins also affects their mechanical properties, where the elliptical-like structure has better wind resistance [13]. Liu [14] tested the flight trajectory of P by multiple high-speed cameras and found that the wing tips had a trajectory similar to the shape of an “8”, and the left and right wings did not move in the same trajectory during flapping, although the flapping phase was the same, and these characteristics indicated that the beetle could flexibly adjust its hind wings to obtain the best aerodynamic performance during flight.

Currently, researchers usually use simulation to determine the applicability of bionic tests, Abramowicz [15] defined an unsteady transient computational fluid dynamics simulation of a flying bionic organism using ANSYS Fluent to compute aerodynamic forces. The transient CFD (Computational Fluid Dynamics) simulation was used to solve the problem of unsteady flow around the flapping wing. In the field of flapping wing flight, wind tunnels are often an effective tool used by many scholars to study the laws of flapping wing flight and aerodynamic characteristics [16,17,18]. When studying the flapping motion and aerodynamic characteristics of organisms in nature, researchers usually choose low-speed straight-flow wind tunnels to conduct qualitative studies on insects or birds [19,20,21,22,23]. To improve the flight performance of flexible film flapping wings, Nian [23] designed a flexible flapping wing that can attach an airfoil from the root, tested it in a wind tunnel, and obtained the performance differences of different airfoils and different thickness flapping wings. Researchers from Brown University and Columbia University [24,25] jointly designed a four-degree-of-freedom bionic flapping wing based on bat wings as the bionic prototype, tested its lift, drag, and energy consumption in the hovering flight state through a wind tunnel test, and provided the results of the kinematic test of the multi-degree-of-freedom flapping wing.

Current biomimetic studies on beetles mainly focus on the mechanical properties of the hind wings and the influence of the hind wing structure on their flight characteristics [26], and the analysis of the aerodynamic characteristics of the deployable hind wings of beetles and the design of the biomimetic deployable wings need to be further studied [27]. In this paper, a bionic deployable wing was designed according to *P. brevitarsis* hind wing structure elements and simulation results, and the multiple factors affecting the aerodynamic characteristics of the bionic deployable wing were investigated through simulation and optimization of the wind tunnel test to obtain the best combination of wind tunnel test parameters. The structural statics and aerodynamic characteristics of the bionic deployable wing were analyzed by finite element simulation. Combined with previous simulation tests, by setting different wind tunnel test parameters, the change curves of lift, drag, and lift-to-drag ratio of the bionic folding wing under different wind speeds, flapping frequencies, and angles of attack are obtained. The aerodynamic characteristics of the bionic folding flapping wing are analyzed, which provides technical support for the wing design of bionic folding flapping wings for MAVs.

## 2. Materials and Methods

### 2.1. Beetle

Six *P. brevitarsis* were selected as research subjects. The adult is nocturnal and has strong flying ability, false death, and phototaxis [14]. The average measured hind wing length of the six beetles is 26.47 ± 1.36 mm, the average width is 8.64 ± 0.54 mm, the average hind wing area is 181.34 ± 15.35 mm^2^, and the aspect ratio (the square of the length of the wing divided by the area of the wing) is 3.86. According to reference [28], the folding ratio is calculated as the ratio of the area of the unfolded and folded hind wings, and according to this algorithm, the beetle hind wing folding ratio is 2.8. The folding ratio calculation method in this paper focuses more on the ratio of the length of the hind wings before and after folding, as the folded length of the wings were divided by the original length, and according to this calculation method, the beetle hind wing folding ratio is calculated as 0.44. The *P. brevitarsis* used in this experiment were collected from Changchun, Jilin Province. All insects were adapted under standard laboratory conditions (ventilation room, 25 ± 1 °C, 60 ± 5% humidity, 12 h light/dark cycle) and had free access to standard water and food. All procedures were conducted in accordance with the Guidelines for Animal Care and Use (China) and approved by the Laboratory Animal Welfare Ethics Committee of Jilin University.

### 2.2. Geometric Model

According to the bionic elements of the folding/unfolding mechanism of the hind wing of *P. brevitarsis* and combined with the research results of the hind wing surface/profile structure and aerodynamic characteristics, the design of the bionic deployable wing was conducted. Biomimetic elements of the hind wings of *P. brevitarsis* are described below.

#### 2.2.1. Vein Structure

The vein profile of the hind wings of *P. brevitarsis* is elliptical, and each wing vein is not uniform in thickness and is of variable diameter [29]. This structure provides support for *P. brevitarsis* to spread its hind wings during flight while improving the torsional resistance of its hind wings [13]. Considering the preparation of bionic deployable wings and the accuracy of 3D printing materials, the wing veins of bionic deployable wings are designed as solid variable-diameter structures.

#### 2.2.2. Hind Wing Folding Method

The folding method of the hind wings of *P. brevitarsis* is a transverse V-shape, which improves the folding ratio of the hind wings, effectively reduces the wing area, and facilitates the miniaturization design of bionic foldable wings [29].

**Figure 1:**
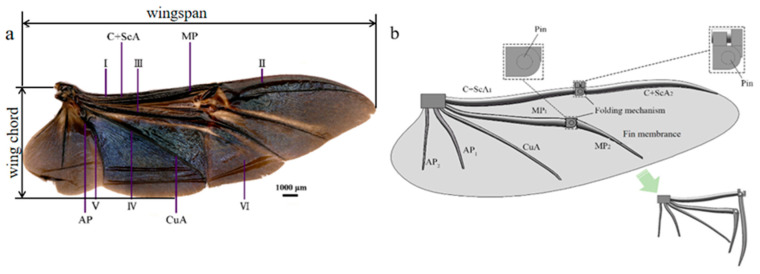
Comparison of (**a**) beetle hind wings and (**b**) bionic wings.

Figure 1a,b show the comparison of the beetle hind wing and the bionic wing. The designed bionic deployable wing mainly consists of wing veins, wing membrane, folding mechanism, and cylindrical pins, as shown in Figure 1b. The wing veins retain the main trunk wing veins of the hind wings of *P. brevitarsis*, which are the costae vein (C+ScA_1_, the front end of the costae vein, C+ScA_2_, the backend of the costae vein), posterior median (MP_1_, the front end of the posterior median, MP_2_, the backend of the posterior median), cubital vein (CuA), and gluteal vein (AP_1_, AP_2_). The folding section uses a rotating sub-mechanism connected by a cylindrical pin, and the deployable wing requires an external force to fold and deploy. Figure 1b shows the unfolded and folded state of the bionic deployable wing veins. Bionic deployable wing folds in a V-fold pattern with an FR of 0.44 and an aspect ratio of 3.86.

The bionic deployable wing veins are all elliptical solid structures. Due to the inhomogeneity of the wing vein diameter, only the diameter parameters of the first and end sections of the different wing veins (elliptical major axis and minor axis) are provided here. The material properties of the wing veins are set to the material properties of high-performance nylon, with an elastic modulus of 1.31 GPa, a Poisson’s ratio of 0.3857, and a density of 1.08 g/cm^3^. The dimensions of the wing vein structure are shown in Table 1 (where C_1_ represents the front cross-section, C_2_ represents the end cross-section, D_maj_ represents the diameter of the major axis, and D_min_ represents the diameter of the minor axis). The material property of the fin membrane is set to Polyester film (PETP), which has a thickness of 0.025 mm, an elastic modulus of 4 GPa, a Poisson’s ratio of 0.36, and a density of 1.389 g/cm^3^. The hind wing length of the bionic deployable wing is set to 112.32 mm, and the hind wing width is set to 34.27 mm. The gravitational acceleration is 9.807 m/s^2^.

### 2.3. Analysis of Mechanical Properties of BD-W

#### 2.3.1. Static Analysis

**Figure 2:**
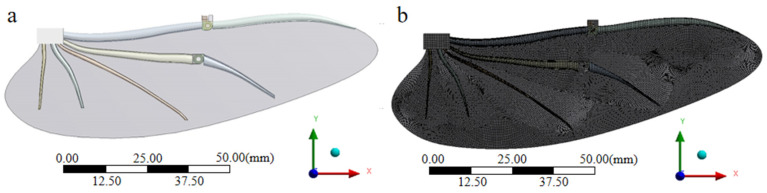
(**a**) Bionic deployable wing finite element model; (**b**) bionic deployable wing finite element model meshing.

The finite element model of the bionic deployable wing is shown in Figure 2a. After assigning the respective material properties to the model in Engineering Date, the model was opened in Geometry. Each wing vein and fin membrane of the bionic deployable wing were meshed respectively in Mech. The mesh division format is based on hexahedra, and tetrahedron and hexahedra are mixed with each other, and the mesh of the wing vein part is refined separately, and the minimum cell size of the refined mesh is 0.2 mm. The overall number of cells in the model is 281,476 and the number of nodes is 1,297,235. The final modified mesh division is shown in Figure 2b.

Applying torque to the leading-edge vein. Fixing the position of the base of the bionic deployable wing, limiting all its degrees of freedom, the torque is applied to the costae vein with a size of T = 3.37 × 10^−1^ N·mm, the calculation formula is as follows:

(1) G=mg

(2) $$F=\frac{1}{2}G$$

(3) $$T=\frac{F}{2}\times \frac{C_{w}}{2}$$

where: *m* is the mass of the bionic deployable wing vehicle model; *C_w_* is the chord length of the wing; and the overall displacement deformation and equivalent force distribution of the bionic deployable wing model are analyzed.

Torque is applied to the entire bionic deployable wing model. Fixing the fin base position and limiting all its degrees of freedom, a uniform load of size 4.65 × 10^−2^ N is applied to the whole hind wing of the bionic deployable wing model with the direction perpendicular to the hind wing downward. The calculation equation is shown in (2) to analyze the overall displacement deformation and equivalent force distribution of the bionic deployable wing model.

#### 2.3.2. Aerodynamic Characteristic Analysis

In this paper, Fluent is used to carry out fluid simulation of the bionic deployable wing to explore its aerodynamic characteristics under different angles of attack, flapping frequency, and wind speed, and to analyze its lift, drag coefficient, and the variation of the surrounding flow field.

(1) Bionic deployable wing aerodynamic model

**Figure 3:**
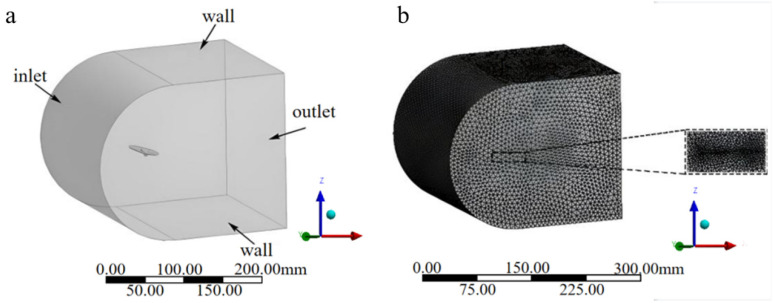
(**a**) Flow field and bionic deployable wing aerodynamic model; (**b**) overall flow field mesh and internal local enlargement.

Using the Fluent module in ANSYS Workbench 19.2, the bionic deployable wing is pre-processed in Geometry, the model is pre-processed geometrically, and the flow field is divided, respectively. The flow field model is shown in Figure 3a, where the flow field is 300 mm long, 200 mm wide, and 200 mm high. The left side of the flow field is the inlet, the right side is the outlet, and the upper, lower, front, and rear sides are the static wall walls. In this paper, a single bionic deployed wing model is used for the fluid simulation. In order to ensure the correctness and accuracy of the calculation results, the bionic deployable wing model is placed at a moderate position within the flow field not close to the boundary, and its position is close to the velocity inlet, which can reduce the influence of the boundary on its return flow. The model is imported into Fluent–Mesh module for meshing. In this paper, tetrahedral meshing is performed for the flow field and the model, respectively, with different mesh cell sizes, and the model is also encrypted. The final meshed model is shown in Figure 3b, where the number of generated mesh cells is 2,401,599 and the number of nodes is 434,285. More than 90% of the grid cell quality is above 0.5, and more than 90% of the grid distortion rate is below 0.5, so the grid quality is good enough to meet the computing needs.

(2) Parameterization of fluid mechanics simulation

The meshed computational domain model is imported into the Fluent–Setup solver for solving parameters setting. Before setting the solution parameters, the number of cores, accuracy, and unit system of the solver should be set. In this paper, 16 cores with double accuracy are used for the solution, and the unit size is mm.

Set boundary conditions:

1. Inlet boundary: The velocity inlet boundary is selected, and the velocity value size is set according to the test.
2. Outlet boundary: The pressure outlet boundary is selected, and the outlet pressure value is set to standard atmospheric pressure.

According to the formula of Reynolds number:

(4) $$Re=\frac{\rho_{\alpha }\nu C_{L}}{\mu }=\frac{2\rho_{\alpha }f\Phi l^{2}}{\mu \lambda }$$

where: *Re* is the Reynolds number; *ν* is the wingtip speed; *ρ_α_* is the air density:1.225 kg/m^3^; *Φ* is the flapping amplitude: 90°; *C_w_* is the chord length: 28.95 mm; *l* is the wingspan length: 2.32 mm; and *μ* is the viscosity of air: 1.789 × 10^−5^ kg/(m·s).

The calculated Reynolds number of the model in flight is 6994.52, which is in the low Reynolds number range. For the bionic deployable wing designed in this paper, the air density around the wing can be considered constant due to the small size of the wing and the low flight speed, so the pressure-based solver is selected. In addition, the flapping process of the wing is a non-constant flow, so the transient solver is selected. Combining with the previous experience and the low Reynolds number calculation, the k-ε SST model under the classical V-L turbulence model is finally chosen in this paper, which is mainly used for the low Reynolds number flow calculation and has greater calculation accuracy. After many tests, the model has a good fit with the simulation test.

In this study, the use of the dynamic mesh technique with UDF equations allows for controlling of the flapping motion of the model in the computational domain for numerical analysis. The motion of the bionic deployable wing is a single-degree-of-freedom flapping motion and its equations of motion are as follows:

*Φ* = *Φ*_0_sin(2πft) (5)

where: *Φ*_0_ is the fixed flapping amplitude; *f* is the flapping frequency.

The values of the simulated parameters for different wind speeds, angles of attack, and flapping frequencies set in this test are shown in Table 2:

Where the flapping angle is the total angle of the wing in the flapping plane, and the angle of attack is the angle between the stroke plane and the horizontal inflow direction.

(3) Aerodynamic characterization of bionic deployable wing

During the flapping of the wings, the airflow will flow over the wings to make them subject to the force. The component force on the wings in the direction perpendicular to the incoming velocity is called the lift force, expressed by *F_L_*, and the component force parallel to the incoming velocity is called the drag force, expressed by *F_D_*. For different conditions of lift and drag, the dimensionless numbers of lift coefficient *C_L_* and drag coefficient *C_D_* are generally used to express, and the definitions of lift coefficient C_L_ and drag coefficient *C_D_* are as follows:

(6) $$C_{L}=\frac{F_{L}}{0.5\rho \nu^{2}S}$$

(7) $$C_{D}=\frac{F_{D}}{0.5\rho \nu^{2}S}$$

where: *F_L_* is the lift force; *F_D_* is the drag force; *ρ* is the air density; *ν* is the wingtip speed; and *S* is the wing area.

### 2.4. Fabrication of BD-W and Wind Tunnel Test

**Figure 4:**
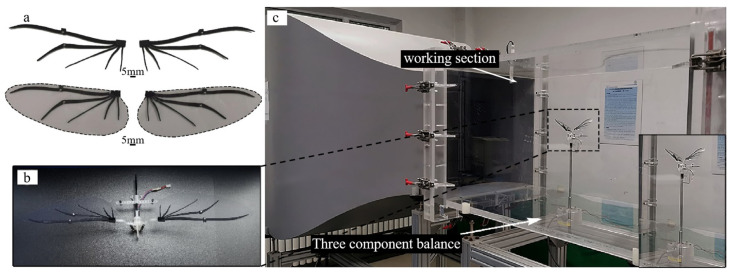
Bionic deployable wing wind tunnel test: (**a**) bionic deployable wing veins and bionic deployable wing assembly; (**b**) flapping wing system; (**c**) installation diagram.

The wing vein material of the bionic deployable wing should have good fatigue resistance, high strength, and high toughness. Considering the accuracy of current 3D printing materials and the properties of the material itself, the wing vein of the bionic deployable wing designed in this paper is made of high-performance nylon material. The fin membrane material of the bionic deployable wing should meet the characteristics of toughness, folding resistance, and ability to withstand high-frequency flapping. Considering that the thickness of the bionic deployable wing membrane should be as close as possible to that of the beetle’s hind wings, polyethylene film (PETP) with great overall performance was chosen as the wing membrane material. The bionic deployable wing manufactured by processing is shown in Figure 4a, and the bionic deployable wing veins and the complete assembly of the bionic deployable wing are shown respectively. The wings of the bionic deployable wing are all elliptic solid radial structures. The single wing length is 112.32 mm, the FR is 0.44, the aspect ratio is 3.88, and the weight of the final assembled single wing is 1.13 g.

The flapping wing system used in the test is shown in Figure 4b. The flapping wing system consists of bionic deployable wing and flapping wing transmission mechanism, which was designed by the research group in the early stage [14]. The mechanism is made of high-performance nylon material and manufactured by 3D printing technology. The flapping mechanism only performs flapping motion, and the driving mechanism adopts gear-reduction mechanism and is controlled by micro DC (direct current) motor, which can realize flapping frequency of ~0–12 Hz. When the flapping frequency is 12 Hz, the motor voltage at this time has not reached the rated voltage, but the motor continues to run at large voltage due to temperature increase and brush wear, resulting in a slow decrease in performance, so the test range of the bionic deployable wing flapping frequency is set to ~0–10 Hz. In addition, the structural design of this flapping drive mechanism is based on *P. brevitarsis* as the prototype, and its flapping angle can realize ~0–90° flapping motion, which accords with the designed bionic deployable wing and the wind tunnel test. The total weight of the assembled flapping system is 9.48 g.

During the wind tunnel test of the bionic deployable wing, the force perpendicular to the direction of incident velocity and the force parallel to the direction of incident velocity were recorded under different parameters, and the lift-to-drag ratio was obtained after filtering and calculation to analyze the aerodynamic characteristics.

The wind tunnel test was performed in a low-speed straight-flow wind tunnel at the Key Laboratory of Bionic Engineering, Jilin University, China. The main parameters of the wind tunnel are shown in Table 3. The test model is first fixed on the stand, which is connected to the force balance (load cell) after adjustment. The selected load cell (LH-SZ-02, Shanghai Liheng, Shanghai, China; 0–20 N ± 0.2 mN) has the advantages of small size, high precision, and fast response, suitable for the flight performance tests of insects and MAVs. As shown in Figure 4c.

Combined with the preliminary simulation test, the wind tunnel test parameters of the final design are the same as Table 2. Then, 0 m/s is added in the column of wind speed parameters as a control. In order to reduce the error of the test results and ensure accuracy, the measurement was recorded three times under each parameter combination, and each flapping time was 10 s. The experimental results were based on the average lift and lift-to-drag ratio as the optimization criteria of aerodynamic performance.

## 3. Results and Discussion

### 3.1. Statics Mechanism Analysis of the Designed BD-W

**Figure 5:**
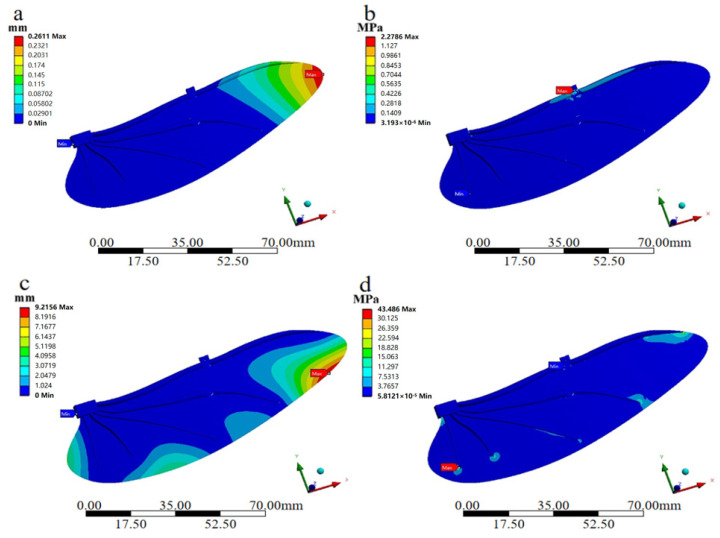
Static analysis diagram of torque acting on the costae vein: (**a**) overall displacement deformation diagram; (**b**) equivalence force diagram. Static analysis diagram of load applied to the entire hind wing: (**c**) overall displacement deformation diagram; (**d**) equivalence force diagram.

The overall displacement deformation diagram and equivalent force diagram of the bionic deployable wing model are shown in Figure 5. Figure 5a,b show the overall displacement deformation diagrams and equivalent force diagrams of the bionic deployable wing model under the torque acting on the costae vein, respectively. As shown in Figure 5a, under the action of torque, the structural deformation of the bionic deployable wing model was concentrated at the right end of the model and gradually decreases inward, with the smallest deformation at the root position. The maximum deformation position appears at the wing tip position, and the maximum deformation is only 0.2611 mm, which indicates that the bionic deployable wing has good torsional resistance. As shown in Figure 5b, the load of the bionic deployable wing model was basically carried by the costae vein, and the stress around the wing was small, while the stress on the costae vein was large and appears to be concentrated, indicating that the costae vein provides support and stress relief for the whole rear wing. The maximum stress appears in the folding part, which is 2.786 MPa, and it also shows that the load on the folding part of the bionic deployable wing was larger compared with other parts, so in the actual design, higher-strength materials should be used to improve the structural stiffness of the folding part.

Figure 5c,d show the overall displacement deformation and equivalent force diagrams of the bionic deployable wing model with the load applied under the whole folded wing, respectively. From Figure 5c, it can be seen that after the model was subjected to uniform vertical downward load, the deformation variables at different locations of the hind wing were different, where the larger deformation locations all appeared on the posterior edge of the wing membrane, and the deformation on the wing vein was smaller, with the maximum deformation reaching 9.2156 mm, which was caused by the lesser number of wing veins on the posterior edge. This indicates that the wing veins provide great support, and at the same time, the deformation on the wing membrane is larger, indicating that the bionic deployable wing will suffer larger passive deformation in practical application, so the wing membrane material with higher elastic modulus and better toughness should be selected in the actual design to avoid structural damage. From Figure 5d, it can be seen that after the model is subjected to uniform vertical downward load, its stresses are more concentrated at the end of each wing vein, where the maximum stress reaches 43.486 MPa. This shows that the end of the fin vein and the fin film connected part of the load is larger, the higher the stress value means that its location is more likely to be destroyed, the structural strength of the wing vein and the fin membrane connected part is weaker, so in the design and actual assembly should pay attention to the connected parts of the cooperation, as well as the use of higher strength materials to improve the resistance to damage here. Comparing the wing veins and the folding parts, it is found that they were not subjected to significant load, which indicates that the uniform load has a small impact on the overall structure of the bionic deployable and the effect is not significant, and the designed bionic deployable wing has good overall structural stiffness.

### 3.2. Aerodynamic Characteristics of BD-W

#### 3.2.1. The Influence of Incoming Flow Velocity

**Figure 6:**
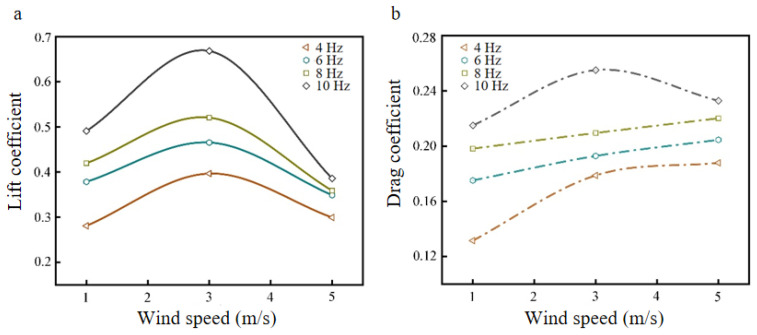
Lift coefficient curve and drag coefficient curve at different wind speeds: (**a**) lift coefficient curve; (**b**) drag coefficient curve.

Setting the flapping angle to 90° and the angle of attack to 15°, the lift coefficient CL and drag coefficient CD curves of the bionic deployable wing model with wind speed at different flapping frequencies were obtained, as shown in Figure 6.

Figure 6a shows the lift coefficient curves of the rigid wing model with wind speed at different flapping frequencies, and it can be seen from the figure that with the increase of wind speed, the lift coefficients at each flapping frequency show a trend of increasing and then decreasing. At low-flow velocities (0–3 m/s), the lift coefficients of the model gradually increase and reach the maximum, and when the wind speed continues to increase above 3 m/s, the lift coefficients begin to show a rapid decrease and gradually converge to a similar level, which is because the flow velocity at this time destroys the stability of the flow field around the wing, and the instantaneous lift cannot be effectively captured [30]. Comparing the variations of lift coefficients at different flapping frequencies, from 1 m/s to 3 m/s wind speed, it is found that the increase of lift coefficient was more obvious when the flapping frequency was 10 Hz, which increased by 36.05% compared to that at 1 m/s wind speed. In addition, it is found that the effect of different flapping frequencies on the lift coefficient was also significant, with the maximum lift coefficient occurring at a flapping frequency of 10 Hz, reaching 0.67. As shown in Figure 6b, the drag coefficient curves of the model with wind speed at different flapping frequencies are shown, and it can be seen from the curve trend that with the increase of wind speed, the drag coefficient at each flapping frequency shows a gradual increase (except 10 Hz). The drag coefficient at high-flapping frequency (10 Hz) showed a trend of increasing and then decreasing, which indicates that the drag coefficient does not always increase with the increase of wind speed. In addition, when the wind speed increased to more than 3 m/s, it is found that the drag coefficient increased slowly at a low-flapping frequency and decreased slowly at a high-flapping frequency, which indicated that the effect of the drag coefficient was not more obvious at high wind speed wing. On the other hand, comparing different flapping frequencies (4 Hz, 6 Hz, 8 Hz, 10 Hz), the change of drag coefficient at ~1 m/s–5 m/s wind speed was found to increase by 42.89%, 16.79%, 11.10%, and 8.28%, respectively. This indicates that with the increase in wind speed, the effect on the drag coefficient of low-flapping frequency was more obvious, and the increase was larger. In summary, the best-simulated parameter value of wind speed for this model is 3 m/s.

#### 3.2.2. The Effect of Flapping Frequency

**Figure 7:**
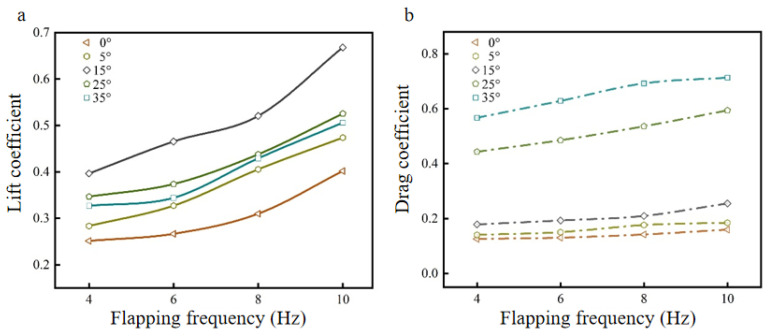
Lift coefficient curve and drag coefficient curve at different flapping frequencies: (**a**) lift coefficient curve; (**b**) drag coefficient curve.

Set the wind speed to 3 m/s and change different flapping frequencies to obtain the lift coefficient CL and drag coefficient CD curves of the bionic deployable wing model that change with flapping frequencies at different angles of attack, as shown in Figure 7.

As shown in Figure 7a, the lift coefficient curves of the model with the change of flapping frequency at different angles of attack are shown, and the trend of the curves shows that the lift coefficient at each angle of attack gradually increases with the increase of flapping frequency. Comparing the changes of lift coefficients at different angles of attack (0°, 5°, 15°, 25°, 35°) with flapping frequencies from 4 Hz to 10 Hz, it is found that it increased by 59.82%, 67.15%, 68.46%, 51.63%, and 54.76%, respectively. This indicates that the lift coefficient is greatly affected by the flapping frequency, and the increase in the flapping frequency helps to improve the lift coefficient. The size of the flapping frequency is generally controlled by the driving mechanism of the flapping system; the higher the flapping frequency, the higher the energy consumption is required, so the flapping frequency cannot be increased indefinitely, within the range of the flapping system, the flapping frequency can be increased appropriately to improve the aerodynamic performance effectively. Comparing the lift coefficients of different angles of attack at the same flapping frequency, it is found that the model showed great aerodynamic performance at 15° angle of attack, which increased by 57.76%, 74.66%, 67.95%, and 66.28% at different flapping frequencies (4 Hz, 6 Hz, 8 Hz, and 10 Hz) compared to 0° angle of attack. As shown in Figure 7b, with the drag coefficient curves of the model under different angles of attack with the change of flapping frequency, from the curve trend, it can be seen that with the increase of flapping frequency, the drag coefficient under each angle of attack also shows a gradual increase trend. Comparing the change of drag coefficient for different angles of attack (0°, 5°, 15°, 25°, 35°) at flapping frequencies from 4 Hz to 10 Hz, it is found that it increased by 23.74%, 31.55%, 42.70%, 34.06%, and 25.75%, respectively, which is a smaller increase compared to the lift coefficient. This indicates that an increase in flapping frequency has a less pronounced effect on the drag coefficient than the lift coefficient, but it does result in a slow increase in the drag coefficient. The increase in drag coefficient indicates that more power is required to overcome the drag. Comparing the drag coefficients of different angles of attack at the same flapping frequency, it is found that the drag coefficient increased with the increase of the angle of attack, and in addition, the drag coefficient at low angles of attack (≤15°) was significantly smaller than that at high angles of attack. The drag coefficient increases by 59.87% at ~0–15° angle of attack and 179.61% at ~15–35° angle of attack for a flapping frequency of 10 Hz, which is due to the destruction of the flow field structure at the angle of attack greater than 15°. As the flapping frequency increases, the lift coefficient also increases. Although the drag coefficient increases at a slower rate, the lift-to-drag ratio reaches its maximum at 10 Hz. In summary, the optimal parameter value for the model flapping frequency is 10 Hz.

#### 3.2.3. The Effect of the Angle of Attack

**Figure 8:**
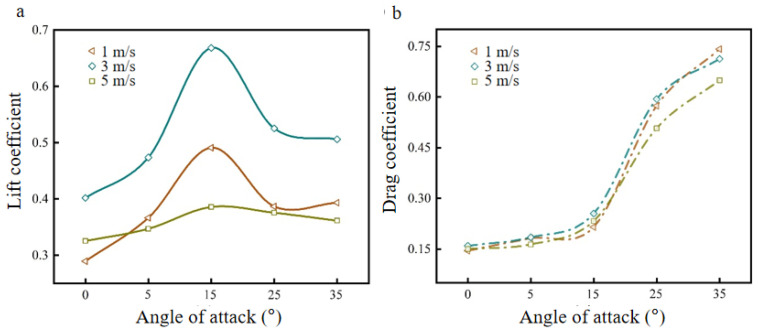
Lift coefficient curve and drag coefficient curve at different angles of attack: (**a**) lift coefficient curve; (**b**) drag coefficient curve.

Setting the flapping frequency at 10 Hz, the lift coefficient CL and drag coefficient CD curves of the bionic deployable wing model were obtained with the change of angle of attack at different wind speeds, as shown in Figure 8.

As shown in Figure 8a, the lift coefficient curves of the model with the change of angle of attack at different wind speeds, the lift coefficients at each wind speed show a trend of first increasing and then decreasing. At a 15° angle of attack, the lift coefficient peaked at each wind speed, with the largest peak at 3 m/s wind speed. Comparing the variation of lift coefficients of the model at different wind speeds (1 m/s, 3 m/s, 5 m/s) from a 0°-to-15° angle of attack, it is found that it increased by 69.57%, 66.28%, and 18.57%, respectively, compared to 0°. This shows that with the increase of the angle of attack, the effect is more obvious, and the increase is large for the case of low-flow velocity. When the angle of attack is greater than 15° and continues to increase, it is found that the lift coefficient decreases faster at this time, which is due to the insignificant increase in the area of the positive and negative pressure zones on the upper and lower airfoils of the model and the decrease in the force surface in the direction of lift, which in turn leads to a rapid decrease in the lift coefficient [31]. This indicates that the angle of attack contributes to the lift coefficient within a certain range, but it does not increase the angle of attack indefinitely. Figure 8b shows the drag coefficient curves of the model with the change of angle of attack at different wind speeds, and it can be seen from the curve trend that the drag coefficients at each wind speed show a gradual increase with the increase of the angle of attack. This is because the contact surface between the model airfoil and the airflow increases, and the drag force is increased. Comparing the changes of drag coefficients at different wind speeds (1 m/s, 3 m/s, 5 m/s), it is found that the drag coefficients increased by 48.28%, 59.87%, and 54.99% at 0~15° angle of attack, and by 245.12%, 179.61%, and 179.04% at a ~15–35° angle of attack, respectively. When the angle of attack is greater than 15° and continues to increase, it is found that the drag coefficient increases rapidly at this time, which is because the excessive angle of attack makes the vortex generated by the flapping wing break up, the flow field structure is disturbed, and the range of wingtip vortex strength increases to affect the surface of the airfoil, resulting in an increase in the surface drag, a rapid increase in the drag coefficient, and a decrease in the aerodynamic performance of the flapping wing [31]. Comparing the overall drag coefficient changes of the angle of attack from 0° to 35° at different wind speeds, it is found that they all have a large increase, which indicates that the effect on the drag coefficient is significant with the increase of the angle of attack, so a suitable range of the angle of attack should be selected to ensure the best aerodynamic performance is obtained. In summary, the best parameter value for the model angle of attack is 15°.

#### 3.2.4. Lift-to-Drag Ratio at Different Parameter Values

**Figure 9:**
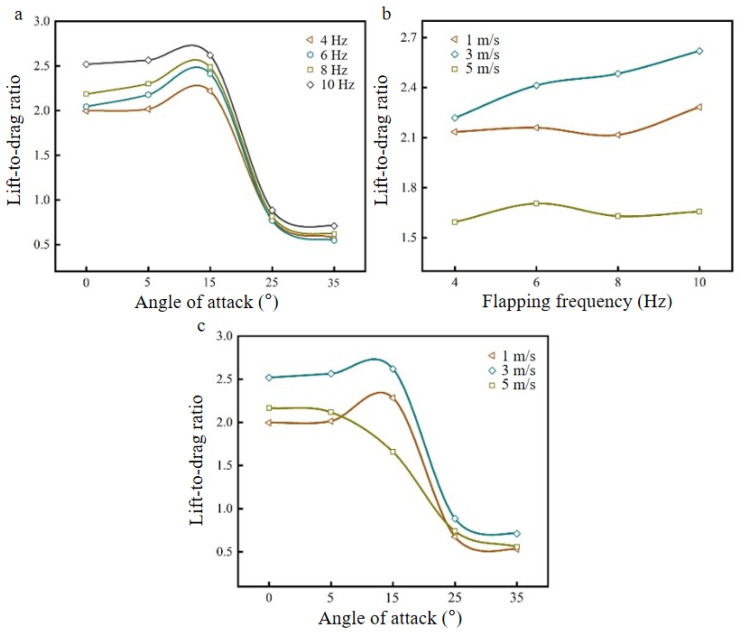
K values under different parameter values: (**a**) K-curve with angle of attack at different flapping frequencies (wind speed of 3 m/s); (**b**) K-curve with flapping frequency at different wind speeds (angle of attack of 15°); (**c**) K-curve with angle of attack at different wind speeds (flapping frequency of 10 Hz).

The lift-to-drag ratio is the ratio of lift and drag or lift coefficient and drag coefficient, expressed as K, and it is an important parameter to evaluate the aerodynamic characteristics of the aircraft and measure the aerodynamic efficiency. The magnitude of K represents the aerodynamic efficiency of an aircraft; the larger the K value is, the higher the aerodynamic efficiency, that is, in the case of the same resistance, the greater the lift generated by the aircraft [32]. Figure 9a–c shows the K curves of the bionic deployable wing model that change with the angle of attack at different flapping frequencies (under a wind speed of 3 m/s), changes with flapping frequency at different wind speeds (under an angle of attack of 15°), and changes with the angle of attack at different wind speeds (under a flapping frequency of 10 Hz).

As shown in Figure 9a, the K value at each flapping frequency tends to increase first and then decrease with increasing angle of attack. At angles of attack of 0–15°, K increases slowly and reaches a peak at 15°. When the angle of attack is greater than 15° and continues to increase, K drops sharply because the average resistance increases monotonically with the angle of attack, and the average lift increases first and then decreases with an increasing angle of attack [33]. This also shows that in a certain range of angles of attack, the increase in the angle of attack helps to increase K; that is, it helps to improve the aerodynamic efficiency. Comparing the K values of different flapping frequencies at an angle of attack of 15°, it is found that the K value at 10 Hz is the largest, which is 2.62.

As shown in Figure 9b, K at each wind speed increases with increasing flapping frequency, but it does not increase gradually, and there is a decline in the middle, which indicates that the change in K is not consistent with the lift coefficient, and it does not increase with increasing flapping frequency. In the case of a high-lift coefficient, K is not necessarily high, so when evaluating the aerodynamic performance of the aircraft, its lift coefficient, drag coefficient, and K value should be comprehensively considered. Compared with the changes in K for flapping frequencies of 4–10 Hz under different wind speeds, the overall trend is upward, which increases by 6.93%, 18.03%, and 3.95%, respectively. This shows that as the flapping frequency increases, it contributes to the improvement in K.

As shown in Figure 9c, K also increases first and then decreases with increasing angle of attack at low wind speeds and gradually decreases at high wind speeds (5 m/s). Compared with the K values of different wind speeds at the same angle of attack, it is found that K at a wind speed of 3 m/s is greater than that of other wind speeds at each angle of attack and reaches the highest value at 15°. When the angle of attack is greater than 15°, K at each wind speed begins to drop sharply and gradually tends to be similar at high angles of attack, which indicates that with the excessive increase in the angle of attack, the flow field structure is disordered at this time, and the influence on K is no longer obvious.

In summary, when the wind speed is 3 m/s, the flapping frequency is 10 Hz and the angle of attack is 15°, K is maximized, and the aerodynamic performance is the best. The lift generated by a single flapping wing under the modified parameter is 6.5 g, which is calculated by formula (5). In this paper, only a single flapping wing is simulated, ignoring the interaction effect of a pair of flapping wings in flight, and the lift generated by a double flapping wing under this parameter is 13 g, which is sufficient to support the bionic deployable flapping-wing vehicle with a mass of 9.48 g.

#### 3.2.5. Simulation Results of BD-W under Optimal Parameters

**Figure 10:**
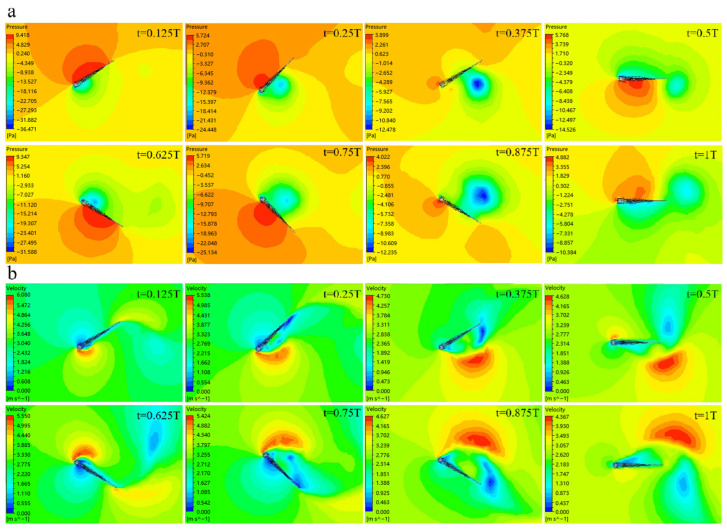
Pressure and velocity contours at different flapping moments: (**a**) Pressure contours; (**b**) Velocity contours.

Figure 10a,b are the pressure-distribution and velocity-distribution contours of the bionic deployable wing model at different flapping moments under the ideal parameters of 3 m/s, 10 Hz, and 15°.

When the wing is in the process of flapping upward (t = 0–0.25 T, t = 0.75–1 T), the airflow velocity at the vortex below the wing surface is much greater than that above, so that the pressure above is stronger than below, resulting in a pressure difference, so that the wing produces negative lift perpendicular to the wing surface in the process of pounce. When the wing is in the process of flapping down (t = 0.25–0.75 T), the air velocity at the vortex above is much greater than that below, and the flow velocity difference between the upper and lower makes the pressure below stronger than the above, resulting in a pressure difference, so that the wing generates lift during the downward flapping. When the wing model reaches the highest point (t = 0.25 T) in the process of pounce, the flapping speed of the wing is very low at this time, mainly for torsional motion. By observing the flow rate around the surface of the wing, it is found that there are high-speed vortices on its leading edge and lower surface. When the wing reaches the middle position (t = 0.5 T) during the downward flapping process, the flapping speed of the wing is the largest, and no torsional movement is performed. At the same time, high-speed vortices appear on its leading edge and under the wing surface, and the leading-edge vortex formed by torsional motion falls off at a farther position below the tail of the wing. When the wing continues to flap and reaches the lowest point (t = 0.75 T), the flapping speed at this time is small, and the torsional movement is mainly carried out. The flow velocity around the wing surface is observed, and high-speed vortices appear near the leading edge and upper surface. When the wing continues to flap upward and reaches the middle position (t = 1 T), the flapping speed at this time is the largest, mainly performing the flapping motion, without the torsion process, and at the same time, there is a high-speed vortex near the leading edge and upper surface, and the leading-edge vortex falls off at a farther position above the tail.

### 3.3. Biomimetic Deployable Wing Wind Tunnel Test

#### 3.3.1. Influence of Incoming Flow Velocity on Aerodynamic Characteristics of BD-W

**Figure 11:**
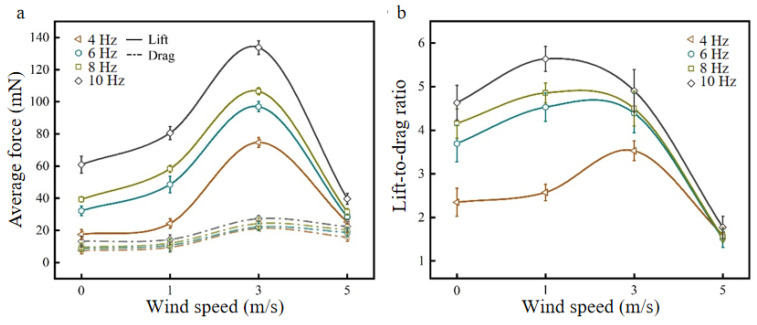
Lift, drag and K curves at different wind speeds: (**a**) lift and drag curves; (**b**) K curve.

The angle of attack of the fixed flapping wing system is 15°, and the wind speeds are changed, as shown in Figure 11. The lift curve, drag curve, and K curve of the bionic deployable wing change with wind speed at different flapping frequencies are obtained.

Figure 11a shows the average lift curve (Figure 11a solid line) and average drag curve (Figure 11a dashed line) of the bionic deployable wing with wind speed at different flapping frequencies. It can be seen from the figure that the lift force under each flapping frequency increases first and then decreases with increasing wind speed, and the lift reaches a maximum at a wind speed of 3 m/s, which is consistent with the simulation results, and at low-flow speed (≤3 m/s), the lift force gradually increases and reaches the peak, and at high-flow speed, the lift force starts to decrease rapidly. Among them, under parameters of 3 m/s and 10 Hz, the lift reaches a maximum of 133 mN, which increases by 119.4% compared with no wind. In addition, it is found that the change in drag is similar to that in lift, and it also increases first and then decreases at each flapping frequency, which is consistent with the trend in the drag coefficient at high frequencies in the simulation results, and the resistance does not increase with increasing wind speed. And, to a certain extent, there is a slow decline. Compared with different flapping frequencies (4 Hz, 6 Hz, 8 Hz, and 10 Hz), the lift and resistance at wind speeds of 0–3 m/s increase, and the increase in resistance is significantly smaller than that of lift, which indicates that within a certain range, an appropriate increase in wind speed can effectively improve lift and improve aerodynamic performance. Under a high wind speed (5 m/s), The lift and drag forces tend to be similar at each flapping frequency, which indicates that the aerodynamic effects of flapping the wing motion are not significant at this point.

Figure 11b shows the average K curve of the bionic deployable wing with wind speed at different flapping frequencies. It can be seen from the figure that K increases first and then decreases as a whole with increasing wind speed, and K reaches a peak at a wind speed of 1 m/s (except for 4 Hz, which reaches a peak at a wind speed of 3 m/s), and the maximum K value occurs at a flapping frequency of 10 Hz, reaching 5.63. The wind tunnel test results are consistent with the simulation results, but the maximum peak in K of the simulation results occurs at a wind speed of 3 m/s, and a wind speed of 3 m/s is taken as the best parameter for the bionic deployable wing after comprehensively considering the changes in lift, resistance, lift coefficient, and drag coefficient.

#### 3.3.2. Influence of the Flapping Frequency on the Aerodynamic Characteristics of BD-W

**Figure 12:**
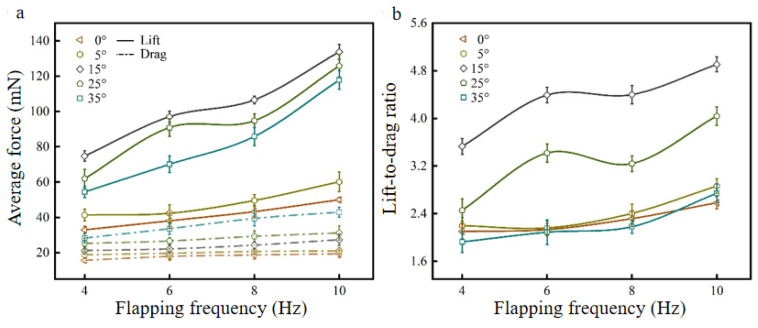
Lift, drag and K curves at different flapping frequencies: (**a**) lift and drag curves; (**b**) K curve.

The fixed wind speed is 3 m/s, and the flapping frequencies are changed, as shown in Figure 12. The lift curve, drag curve, and K curve of the bionic deployable wing change with the flapping frequency at different angles of attack are obtained.

Figure 12a shows the lift and drag curves of the bionic deployable wing at different angles of attack with the change of flapping frequency, and it can be seen from the figure that with the increase of the flapping frequency, the trend of the change of lift and drag at each angle of attack is consistent, and they all show a gradual increase. Comparing different angles of attack (0°, 5°, 15°, 25°, 35°), the lift force at 10 Hz flapping frequency showed a significant increase compared to 4 Hz, where the maximum lift force value appeared at a 15° angle of the attack case, which increased by 79% compared to 4 Hz. In addition, the increase in lift at a high angle of attack (≥15°) is higher than that at a low angle of attack, which indicates that the flapping frequency has a great influence on a high angle of attack. Comparing the change in the drag curve, it is found that the increase is smaller compared to the lift, which is consistent with the simulation results, indicating that as the flapping frequency increases, the effect on the change in lift is greater than the drag. On the other hand, as the angle of attack increases, the overall range of resistance growth becomes significantly large, and the resistance at 10 Hz increases by 23.48%, 28.7%, and 51.97% compared with that at 4 Hz for angles of attack of 0°, 15°, and 35°, respectively.

As shown in Figure 12b, the K values of the bionic deployable wing change with the flapping frequency at different angles of attack, and it can be seen from the curve that with increasing flapping frequency, K under each angle of attack shows an overall increasing trend, but it is not gradually increasing, and there is a decline in the middle, which is consistent with the simulation results, indicating that the change in K is not consistent with the lift and resistance trend, and it is not a guaranteed increase with increasing flapping frequency. The maximum K reaches 4.91 when the working conditions are 15° and 10 Hz, which is an increase of 39.1% compared with 4 Hz. In addition, comparing the changes in K at different angles of attack, it is found that the range of change at low angles of attack is similar, the overall growth range is small, the difference in the range of change at high angles of attack is obvious, and the increase range is large, which indicates that the influence of the flapping frequency on the aerodynamic performance at high angles of attack is more obvious. K is an important parameter for evaluating aerodynamic characteristics, but sometimes it cannot be used as a single reference parameter, and aerodynamic performance should be evaluated in combination with other aerodynamic parameters, such as lift and resistance. Therefore, 3 m/s and 10 Hz are used as the best parameters for this bionic deployable wing.

#### 3.3.3. Influence of the Angle of Attack on the Aerodynamic Characteristics of BD-W

**Figure 13:**
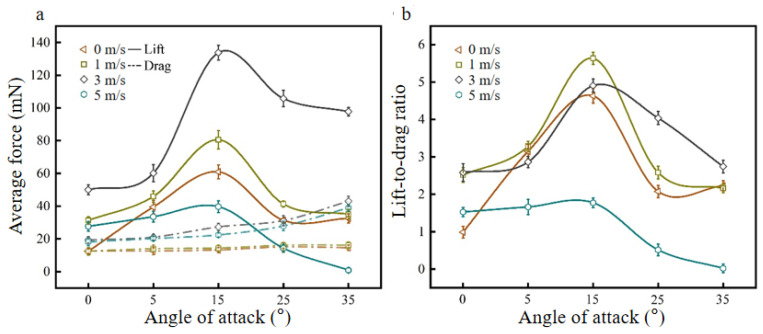
Lift, drag, and K curves at different angles of attack: (**a**) lift and drag curves; (**b**) K curve.

The fixed flapping frequency is 10 Hz, and the angles of attack are changed, as shown in Figure 13. The lift curve, drag curve, and K curve of the bionic deployable wing change with the angle of attack at different wind speeds are obtained.

Figure 13a shows the lift curve and drag curve of the bionic deployable wing with the angle of attack at different wind speeds. It can be seen from the figure that with increasing angle of attack, the lift under each wind speed shows a trend of first increasing and then decreasing. At an angle of attack of 15°, the lift reaches a peak, and the maximum lift appears at a wind speed of 3 m/s. When the angle of attack continues to increase, the lift begins to decrease, which is completely consistent with the simulation results. Comparing the lift change curve for angles of attack of 0–15° t different wind speeds (1 m/s, 3 m/s, and 5 m/s), it is found that with increasing angle of attack, the lift increases and is more obvious at a wind speed of 3 m/s, and the lift at an angle of attack of 15° is 2.68 times that at 0°. Comparing the resistance curves, it is found that the resistance at different wind speeds increases gradually with increasing angle of attack, and the resistance increases relatively significantly at higher wind speeds, which is also caused by the increase in the contact surface between the airfoil and the airflow. In addition, the increase in resistance at low angles of attack (≤15°) is significantly less than that at high angles of attack. At a wind speed of 3 m/s, the resistance at an angle of attack of 15° is 1.41 times that at 0°, and the resistance at 35° is 1.58 times that at 15°. Comparing the overall changes in the lift and drag curves at different wind speeds, it is found that when the angle of attack is greater than 15°, the lift decreases rapidly, the resistance increases significantly, and the lift is less than the resistance at individual wind speeds, which indicates that the flow field structure has been disordered at this time, which has an obvious impact on the aerodynamic performance of the flapping wing.

Figure 13b shows the K curve of the bionic deployable wing with the angle of attack at different wind speeds. It can be seen from the figure that with increasing angle of attack, K at each wind speed also shows a trend of first increasing and then decreasing, and at an angle of attack of 15°, K reaches a peak, which is consistent with the overall change trend of the simulation results. At a high angle of attack, the K decreases significantly, which is caused by an obvious increase in resistance at this time and a decrease in lift. Comparing the K curves for angles of attack of 0–15° at different wind speeds (0 m/s, 1 m/s, 3 m/s, and 5 m/s), it is found that K increases significantly at low wind speeds, while K changes relatively little at high wind speeds, also indicating that the influence of flapping wing motion on aerodynamics at high wind speeds is small.

## 4. Conclusions

According to the structural elements of the hind wing of *P. brevitarsis* and the simulation results, a bionic deployable wing was designed. Using finite element simulation, the structural static and aerodynamic characteristics of the bionic deployable wing were analyzed. Through structural static analysis, under the action of applying torque to the costae vein and applying uniform load to the entire hind wing, the maximum displacement shape variable appeared on the right end of the hind wing and the posterior edge of the wing membrane, respectively, and the stress position appeared at the end of each wing vein, which was more concentrated. Through Fluent software, the aerodynamic characteristics of the bionic deployable wing under different attack angles, flapping frequencies and wind speeds were analyzed, and the lift coefficient, drag coefficient and K of the model under different parameters were obtained. With the constant flapping angle (90°) and angle of attack (15°), the lift coefficients at each flapping frequency showed a trend of increasing and then decreasing with the increase of wind speed, and it reached the maximum value at the wind speed of 3 m/s. The drag coefficient shows a gradual increase with the increase in wind speed (except 10 Hz). The drag coefficient at a high-flapping frequency (10 Hz) shows a trend of increasing and then decreasing, which means that the drag coefficient does not increase with the increase of wind speed. With the constant wind speed (3 m/s), the lift coefficients at all angles of attack showed a gradual increase with the increase of flapping frequency. Comparing the lift coefficients of different angles of attack at the same flapping frequency, it was found that the model shows better aerodynamic performance at 15° angle of attack. The drag coefficient also shows a gradual increase with the increase of flapping frequency, but the increase is smaller compared to the lift coefficient. This indicates that the effect of increasing flapping frequency on the drag coefficient is not as obvious as that of the lift coefficient, but it also leads to a slow increase in the drag coefficient. With the constant flapping frequency (10 Hz), the lift coefficient at each wind speed showed a trend of increasing and then decreasing with the increase of the angle of attack, and the lift coefficient reached the maximum at the angle of attack of 15°. The drag coefficient tends to increase gradually with the increase of angle of attack, which is because the contact surface between the model airfoil and the airflow increases, while the drag force is increased. Comprehensively considering its lift coefficient, drag coefficient, and K, it was found that different parameters impacted the aerodynamic characteristics.

The bionic deployable wings of the design were prepared by 3D printing technology, the wing veins adopted an elliptical solid variable diameter structure, high-performance nylon materials were selected, and the wing membrane adopted a polyester film with great comprehensive properties. Combined with the simulation test, different wind tunnel test parameters were set to obtain the lift, resistance, and K change curves of the bionic deployable wing under different wind speeds, flapping frequencies, and angles of attack, and the aerodynamic characteristics were analyzed. The test results obtained were highly consistent with the simulation results. Combined with the simulation results, comprehensively considering its lift, resistance, K, and other aerodynamic parameters, the best parameter combination was found to be 3 m/s, 10 Hz, and 15°. Under this parameter combination, the aerodynamic performance was the best, and K was as high as 4.91. In future research, the finite element model of the wing veins still needs to be further optimized towards the real beetle hindwing, and motors with similar weight but higher stall torque should be selected to further increase the flapping frequency.

## Figures and Tables

**Table 1:** Vein structure dimensions.

	C+ScA_1_		C+ScA_2_		MP_1_		MP_2_		CuA		AP_1_		AP_2_	
	**C_1_**	**C_2_**	**C_1_**	**C_2_**	**C_1_**	**C_2_**	**C_1_**	**C_2_**	**C_1_**	**C_2_**	**C_1_**	**C_2_**	**C_1_**	**C_2_**
D_maj_, mm	4.20	2.20	2.84	0.80	2.00	2.70	2.70	0.40	1.70	0.75	3.20	0.92	1.60	0.80
D_min_, mm	1.94	1.95	2.09	0.29	1.05	2.00	2.00	0.12	1.20	0.51	1.25	0.34	0.75	0.24

**Table 2:** Simulation parameter values.

Characteristic Parameter	Parameter Range
Flapping angle (°)	90
Wind speed (m/s)	1, 3, 5
Angle of attack (°)	0, 5, 15, 25, 35
Flapping frequency (Hz)	4, 6, 8, 10

**Table 3:** Main parameters of the wind tunnel.

Test Section Parameters	Value
Working section shape	Rectangle
Working section area (mm^2^)	650 × 450
Length of working section (mm)	1000
Turbulent intensity (%)	<0.3
Regulator form of wind speed	Hot-wire sensor
Range of wind speed (m/s)	0–10
Airflow nonuniformity of working section (%)	<3

## Data Availability

Data is contained within the article.
